# Influence of change of tunnel axis angle on tunnel length during double-bundle ACL reconstruction via the transportal technique

**DOI:** 10.1186/s12891-017-1599-9

**Published:** 2017-05-31

**Authors:** Joon Ho Wang, Do Kyung Lee, Sung Taek Chung, Byung Hoon Lee

**Affiliations:** 1 Department of Orthopaedic Surgery, Samsung Medical Center, Sungkyunkwan University School of Medicine, Seoul, 06351 South Korea; 2 Department of Health Sciences and Technology, SAIHST, Sungkyunkwan University, Seoul, 06351 South Korea; 3 Department of Medical Device Management and Research, SAIHST, Sungkyunkwan University, Seoul, 06351 South Korea; 4 Department of Orthopaedic Surgery, Konyang University Hospital, 158, Gwanjeodong-ro, Seo-gu, Daejeon, South Korea; 5 Department of Orthopaedic Surgery, Kang-Dong Sacred Heart Hospital, Hallym University Medical Center, Gil-dong, Seoul, 134-701 South Korea

**Keywords:** Anterior cruciate ligament reconstruction, Transportal, Femoral tunnel orientation, Quadrant method

## Abstract

**Background:**

Commercially available flexible reamer and curved guide systems allow a certain degree of control over intra-articular tunnel orientation, therefore allows a wide range of intra-osseous femoral tunnel orientations, contrary to the femoral tunneling technique using a straight guide pin, which are determined by knee flexion angle. We sought to find the clinical relevance of intra-osseous femoral tunnel orientations in the respect of tunnel length. To evaluate the relationship between the tunnel axis angle in three orthogonal planes and tunnel length in the anteromedial (AM) and posterolateral (PL) femoral tunnels in patients who underwent anatomic double-bundle anterior cruciate ligament reconstruction (DB-ACLR) using the transportal (TP) technique with a 42^o^ curved guide.

**Methods:**

A total of 40 patients who underwent primary DB-ACLR with the TP technique using a curved guide were evaluated retrospectively. The tunnel axis angle in three orthogonal planes were evaluated on a three-dimensional surface model constructed using an axial computed tomography scan obtained after reconstruction. Then, correlations with tunnel length were analyzed.

**Results:**

In the AM tunnel, tunnel axis angles in the coronal (β = 0.0252, *p* = 0.022) and sagittal (β = 0.0168, *p* = 0.029) plane showed significant correlations with tunnel length, while the axial plane did not (*p* = 0.493) (adjusted R^2^ = 0.801). In the PL tunnel, only tunnel axis angles in the axial plane (β = 0.0262, *p* = 0.008) showed a significant relationship with tunnel length (adjusted R^2^ = 0.700).

**Conclusion:**

Drilling at a higher angle in the coronal and sagittal planes in AM tunnels and at a higher angle in the axial plane in PL tunnels decreases the incidence of short femoral tunnels.

**Electronic supplementary material:**

The online version of this article (doi:10.1186/s12891-017-1599-9) contains supplementary material, which is available to authorized users.

## Background

Recently, anterior cruciate ligament (ACL) surgical techniques with a primary focus on anatomic reconstruction have been considered to restore normal knee anatomy, kinematics, and stability more thoroughly [1–3]. This is provided by placing tunnels in the center of native ACL insertion sites on the tibia and femur using either the single- or double-bundle technique [4]. To achieve anatomic ACL femoral tunnel positions more easily, transportal drilling has been proposed as an alternative to the transtibial technique [5–8]. However, more horizontal or oblique femoral tunnel positions result in a shorter distance between the notch and the lateral femoral cortex, which results in shorter overall tunnel length, iatrogenic damage to the medial femoral condyle cartilage [9], and a higher chance of posterior wall blowout from tunneling toward the posterior femoral condyle compared to the traditional transtibial technique [10–12].

Therefore, there are increasing interests to attain an adequate tunnel length and prevent posterior wall breakage [11–13]. Recently, commercially available flexible reamer and curved guide system was made to decrease the chance of injuring the medial femoral condyle cartilage. Furthermore, it has been suggested to achieve longer femoral intraosseous tunnel lengths than with a straight guide pin [14].

**Fig. 1:**
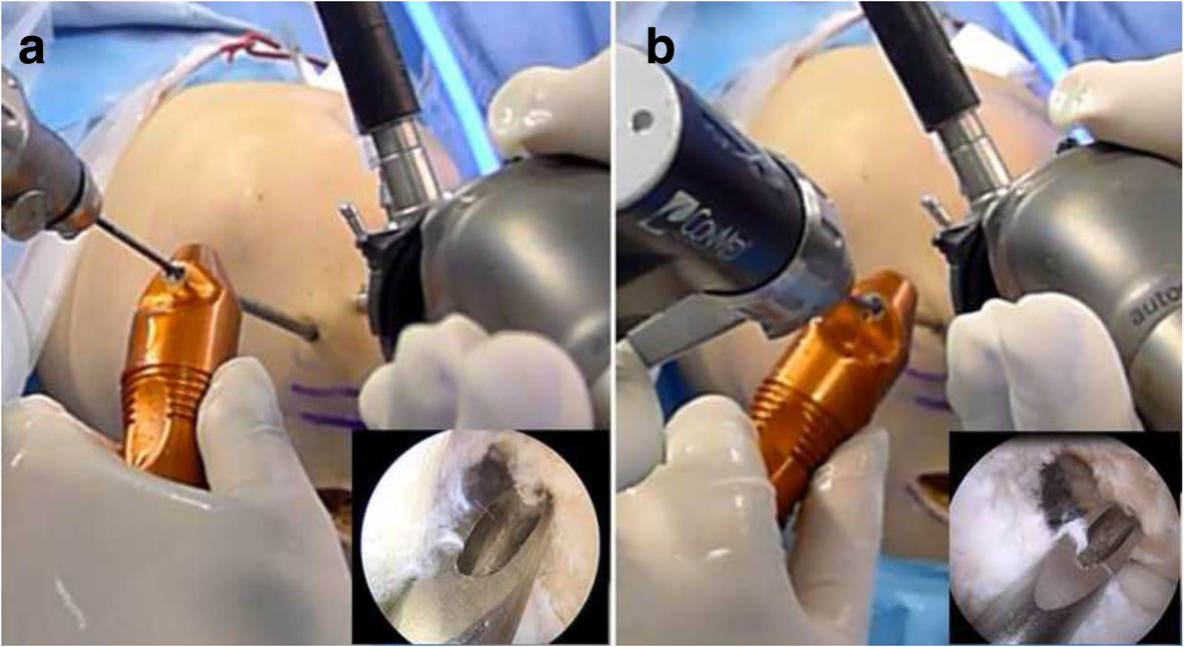
During femoral tunneling procedure using curved guide, the change of intra-articular tunnel orientations could be allowed by the certain degrees of guide’s movement within the intercondylar notch space. **a** First, curved guide is positioned for targeting the intended tunnel position. **b** Then, the guide can be rotated within the intercondylar notch space

**Fig. 2:**
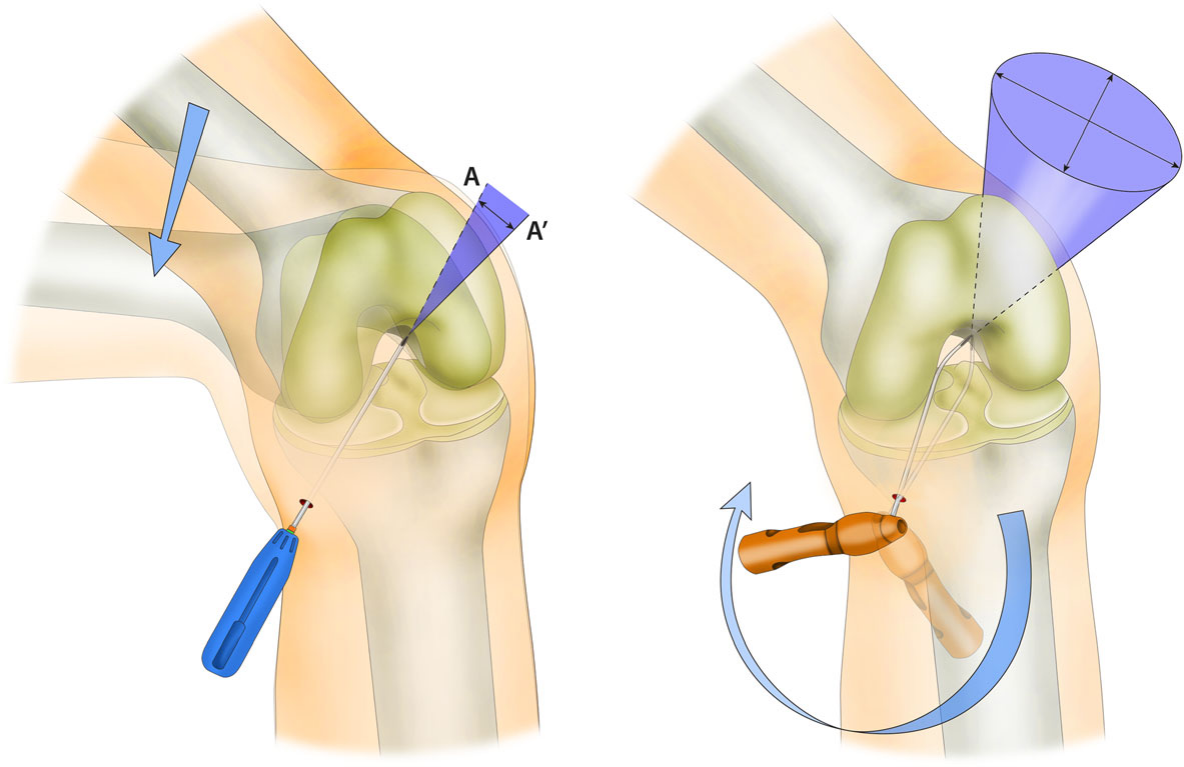
The curved guide system allows a certain degree of control over intra-articular tunnel orientation regardless of the knee flexion angle due to the rotational freedom of the guide in the intercondylar notch space. This is contrary to tunneling with a straight guide pin, which has fixed intra-articular tunnel orientation from two points, the AM or AAM portal and the femoral tunnel center

During femoral tunneling procedure using curved guide, we noticed changeable intra-articular tunnel orientations could be allowed by the certain degrees of guide’s movement within the intercondylar notch space. Meanwhile, in the femoral tunneling technique with a straight guide pin, the intra-articular tunnel orientation is mainly determined by the knee flexion angle because it involves the fixed intra-articular tunnel orientation from two points: the portal, and a femoral tunnel center within the anatomic femoral footprint (Figs. 1 and 2). Controllable intra-articular tunnel orientation can assure a wide range of tunnel axis angles in three-dimensional planes. We became interested in the interosseous tunnel orientation in three dimensional planes, the extension of the intra-articular tunnel orientation.

The purpose of this study was to evaluate the relationship between tunnel axis angle in the three orthogonal planes and tunnel length using in vivo imaging data. The following research question was addressed: In which plane is the tunnel axis angle correlated to the tunnel length in both anteromedial (AM) and posterolateral (PL) femoral tunnels after anatomic double-bundle ACLR? Our hypothesis is that intra-osseous femoral tunnel orientations depending on the entrance angle of the guide pin influence tunnel length, which can be useful for the acquisition of longer tunnel length with use of commercially available flexible reamer and curved guide systems.

## Methods

### Demographic data

Between October 2013 to May 2014, 83 patients underwent primary anatomic double-bundle ACL reconstruction with the transportal (TP) technique using a curved guide and flexible reamer (Clancy Anatomic Cruciate Guide System; Smith & Nephew, Andover, MA, USA) (Table 1).

**Table 1:** Patient demographics and baseline characteristics^a^

	Data
Age, y, mean ± SD (range)	32.3 ± 10.9 (15–57)
Sex, male/female, *n* ^a^	28 / 10
BMI, kg/m^2^	25.4 ± 4.1 (17.6–36.3)
Femoral condyle size	
M-L epicondylar distance (mm)	82.2 ± 5.8 (69.8–95.7)
LPC offset distance (mm)	25.3 ± 2.4 (20.2–29.3)
Time from injury to reconstruction (Log^b^)	1.6 ± 1.9 (−1.6–4.8)

*BMI* body mass index, *M-L* medial to lateral, *LPC* lateral posterior condyle

^a^Values are expressed as mean ± standard deviation (range) except for sex

^b^Time was log-transformed because it showed abnormal distribution

Inclusion criteria were a primary unilateral ACL injury with or without meniscus injury that was treated by double-bundle ACL reconstruction with the TP (accessory AM) technique, as well as patient age ranging from 15 to 60 years. Of the 83 patients, 43 were excluded because they (1) had undergone ACL reconstruction using a rigid guide because there was no flexible reamer size option for either AM or PL grafts less than 6 mm in diameter (*n* = 19), (2) had undergone ACL reconstruction using another technique (outside-in) (*n* = 15), (3) had any combined multiple-ligament injury (*n* = 2), or (4) they had undergone a single bundle reconstruction for open physis (*n* = 2) and simultaneous reconstruction of the ACL and PCL (*n* = 4) combined with HTO (*n* = 1). Finally, 40 patients who underwent anatomic double-bundle ACL reconstruction by the TP technique were retrospectively evaluated in the present study. Institutional Review Board approval (2015-05-085) was obtained from our institution (Samsung Medical Center, Seoul, South Korea) before the study, and the protocol was approved. All patients provided informed consent prior to participation in this study.

### Surgical technique

A single surgeon (J.H.W.) experienced in ACL reconstruction performed all operations using the TP arthroscopic-assisted technique. Femoral and tibial tunnels were created in the centers of their respective anatomic insertions. Grafts were fixed with a cortical suspension system using the shortest possible loop (10 to 15 mm) to ensure maximal contact between the grafts and tunnel walls on the femoral side. Bio-absorbable interference screws with a post tie were used on the tibial side for all cases.

Portal formation was conducted in the usual manner. An anteromedial (AM) portal was placed in a slightly more proximal position than usual, with the distal extent of the portal ending at the level of the inferior pole of the patella. An accessory anteromedial (AAM) portal was made approximately 1.5 cm medial from the standard AM portal and just above the medial meniscus anterior horn. The arthroscope was inserted into the AM portal, and another working device was inserted into the AAM portal. After the ACL rupture was confirmed and remnant tissue was debrided, the femoral footprints of both the AM and PL bundles were carefully defined in reference to the ACL remnants and bony ridges [13]. Centers of both footprints were then indicated with a curved microfracture awl. The center of the AM bundle footprint was 6–7 mm distal (shallow) to the posterior cartilage margin, 2 mm from the posterior bony ridge of the lateral femoral condyle [15, 16], and 3–4 mm posterior (low) to the extended line of the posterolateral corner of the intercondylar notch, which was verified at 90° of knee flexion. The center of the PL bundle footprint was positioned 5 mm anterior (high) to the edge of the joint cartilage on an imaginary line perpendicular to the tangent of the lowermost portion of the lateral femoral condyle at 90° of knee flexion [17]. Our considerations in placement of femoral tunnel were 1) to prevent slippage on the medial wall of the lateral femoral condyle while placing guide wires within the anatomical footprint of the ACL 2) to secure appropriate tunnel length of 20 mm or more 3) to prevent of posterior cortical breakage. After creating the femoral tunnel, its length was measured with a ruler. The required EndoButton size (Smith & Nephew Endoscopy, Andover, MA) for the TP technique was then determined. A femoral guide was inserted through the AAM portal, and a 2.4-mm guide pin was advanced 2 to 3 mm to engage the guide to the center of the AM and PL bundle femoral footprints. The knee joint was then bent as fully as possible and the guide was advanced until the pin passed the cortex and skin. After changing the viewing portal from AM to anterolateral, the tibial footprints of both the AM and PL bundles were carefully defined in reference to the ACL remnants and bony ridges. The anterior margin of the ACL footprint was described as the ACL ridge, and the posterior margin was defined as the retro-eminence of the tibial spine [18, 19].

### Measurement of tunnel axis angle using three-dimensional computed tomography

**Fig. 3:**
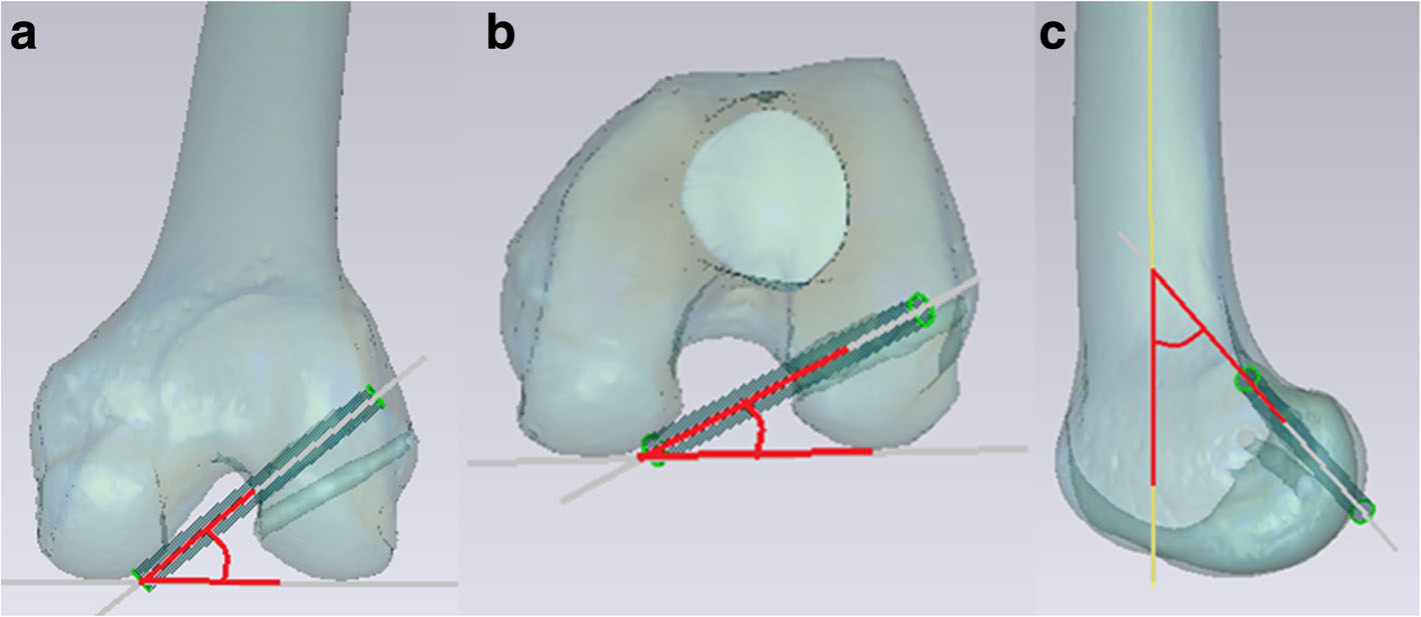
Angle in each plane projected from a three-dimensional surface model. **a** The angle between the tunnel and a line tangent to distal aspects in the coronal plane was measured. **b** The angle between the tunnel and a line tangent to the posterior aspects of the femoral condyles was measured in the axial plane. **c** The angle between the tunnel and the extended intersectional line of the femoral shaft in the sagittal plane was measured

Computed tomography (CT) scans were performed on all knees after ACL reconstruction. The knee was placed in full extension. Digital Imaging and Communications in Medicine data were extracted from the picture archiving and communication system. Data were segmented by Mimics software (Materialise, Leuven, Belgium), a commercially available image processing software used to create three-dimensional (3D) surface models from stacks of two-dimensional image data. The data were then imported into Geomagic Studio software, version 12.0 (Geomagic, Rock Hill, SC, USA), and the 3D surface model was projected into coronal, axial, and sagittal planes to measure the tunnel axis angle in each plane (Fig. 3). As described by Basdekis et al. [20], the angle between the tunnel and the line tangent to the distal and posterior aspects of the femoral condyles was measured in the coronal and axial planes. The sagittal plane angle between the tunnel and the extended intersectional line of the femoral shaft was measured. A lower tunnel angle in the sagittal plane indicated that the tunnel orientation is extended compared with the femoral shaft, while a higher tunnel angle in the sagittal plane indicated that tunnel orientation is flexed compared with the femoral shaft.

To measure the femoral tunnel length, the plane in which the entire length of the femoral tunnel showed the maximal width was selected. The distance between the centers of the intra-articular and extra-articular tunnel apertures was measured [21]. To evaluate its correlation with distal femoral anatomy, the lateral posterior condyle (LPC) AP size (LPC offset distance) and medial-to lateral (M-L) epicondylar distance were measured [21]. The incidence of posterior cortical damage was evaluated by 3D CT scan. Cases with posterior cortical damage or in which the tunnel center was not placed within the anatomical footprint boundary would have been excluded, but none occurred in this study.

## Reliability and statistical analysis

Two orthopedic surgeons (independent observers) together developed and agreed to the measurement methods. They were blinded to each other’s measurements and their own prior measurements. They measured the tunnel axis angle in sagittal, coronal, and axial planes, and tunnel length for AM and PL bundles of all knees. The intra-class correlation coefficient was used to assess the interobserver reliability of measurements.

Uni- and multivariate regression analyses evaluated the relationship between independent influential factors and tunnel length using the SigmaStat software package. Influential factors were considered demographic factors (sex, age, and body mass index), LPC offset distance, and M-L epicondylar distance. Significance was set at *P* < 0.05. All statistical analyses were performed using SAS ver. 9.3 (SAS Institute, Cary, NC, USA).

## Results

The interobserver and intraobserver reliability ranged from 0.83 to 0.96 and 0.85 to 0.95, respectively (Table 2). The mean AM tunnel length was 33.5 ± 4.3 mm (range, 21.6 to 42.9), and the mean PL tunnel length was 35.4 ± 4.0 mm (range, 24 to 41.0).

**Table 2:** Results of Intraclass Correlation Coefficient (ICC) value of each measurement

	AM tunnel			PL tunnel		
	Tunnel Axis Angle in			Tunnel Axis Angle in		
	Coronal plane	Sagittal plane	Axial plane	Coronal plane	Sagittal plane	Axial plane
Intertester						
ICC	0.81	0.85	0.86	0.85	0.91	0.90
Lower ICC	0.67	0.73	0.76	0.74	0.83	0.82
Upper ICC	0.90	0.92	0.93	0.92	0.95	0.95

### Influence of tunnel Axis angle change in 3-D planes on tunnel length in AM and PL femoral tunnels

Tables 3 and 4 show data regarding measured variables.

**Table 3:** Univariate analysis for correlation between femoral tunnel length and independent variables including patient factors^a^

	AM Femoral Tunnel			PL Femoral Tunnel		
Variables	Beta coefficient (β)	Standard error β	*p*-value	Beta coefficient (β)	Standard error β	*p*-value
Age	0.015	0.006	**0.023**	0.005	0.006	0.420
Sex	0.521	0.135	**0.001**	0.647	0.106	**<0.001**
Height	0.038	0.007	**<0.001**	0.040	0.006	**<0.001**
Weight	0.012	0.004	**0.008**	0.014	0.004	**0.001**
Femoral condyle size						
M-L epicondylar distance	0.447	0.100	**<0.001**	0.475	0.086	**<0.001**
LPC offset distance	0.468	0.288	0.112	0.497	0.267	0.072
Tunnel axis angle in						
Coronal plane	0.047	0.010	**<0.001**	0.033	0.009	**0.001**
Sagittal plane	0.001	0.0112	0.933	−0.009	0.004	**0.038**
Axial plane	0.029	0.0088	**0.003**	0.022	0.010	**0.038**

*M-L* medial-to lateral, *LPC* lateral posterior condyle

^a^Values <0.05 are displayed in bold

**Table 4:** Multivariate linear regression analysis for correlation between femoral tunnel length and tunnel axis angle in three dimensional planes^a^ (Entry criteria *p*-value <0.05)

	AM Femoral Tunnel			PL Femoral Tunnel		
Variables	Beta coefficient (β)	Standard error β	*p*-value	Beta coefficient (β)	Standard error β	*p*-value
Tunnel axis angle in						
Coronal plane	0.025	0.010	**0.022**	−0.001	0.012	0.909
Sagittal plane	0.017	0.007	**0.029**	−0.004	0.004	0.331
Axial plane	0.007	0.009	0.493	0.026	0.009	**0.008**

*AM* anteromedial, *PL* posterolateral

^a^Values <0.05 are displayed in bold. Adjusted R square: 0.8012 in AM, 0.6996 in PL femoral tunnel

In the AM femoral tunnel, the mean tunnel axis angle was 44.5^o^ ± 5.7^o^ in the coronal plane, 40.9^o^ ± 6.4^o^ in the sagittal plane, and 32.9^o^ ± 7.2^o^ in the axial plane. Univariate regression analyses identified that the patient factors related to longer femoral tunnel length include male gender (β = 0.5212, *p* = 0.001), age (β = 0.0145, *p* = 0.023), height (β = 0.0375, *p* < 0.001), weight (β = 0.0119, *p* = 0.008), femoral condyle size (M-L epicondylar distance) (β = 0.4473, *p* < 0.001), and greater femoral tunnel axis angle in the coronal (β = 0.0465, *p* < 0.001) and axial (β = 0.0285, *p* = 0.003) planes.

In the PL femoral tunnels, the mean tunnel axis angle was 30.5^o^ ± 6.3^o^ in the coronal plane, 51.7^o^ ± 16.4^o^ in the sagittal plane, and 25.1^o^ ± 6.1^o^ in the axial plane. Univariate regression analyses identified that the patient factors related to longer femoral tunnel length include male gender (β = 0.6471, *p* < 0.001), height (β = 0.0399, *p* < 0.001), weight (β = 0.0136, *p* = 0.001), femoral condyle size (M-L epicondylar distance) (β = 0.4751, *p* < 0.001), and greater femoral tunnel axis angle in all three planes: coronal (β = 0.0326, *p* = 0.001) sagittal (β = −0.0087, *p* = 0.038), and axial (β = 0.0224, *p* = 0.038).

Multivariate regression analysis (Table 4) identified a disparate result between the AM and PL tunnels. In the AM tunnel, coronal (β = 0.0252, *p* = 0.022) and sagittal (β = 0.0168, *p* = 0.029) angles showed a significant correlation with AM femoral tunnel length, while this was not identified in the axial plane (*p* = 0.493) (adjusted R^2^ = 0.801). In the PL tunnel, only the axial angle (β = 0.0262, *p* = 0.008) showed a significant relationship with PL femoral tunnel length (adjusted R^2^ = 0.700).

## Discussion

The principal findings of our study are that tunnel length is correlated with tunnel axis angle in the three-dimensional planes, but differently in AM and PL tunnels. In AM tunnels, tunnel length was related to tunnel axis angle in the coronal and sagittal planes (*p* = 0.022, 0.029 respectively), whereas PL tunnel lengths were related only to the tunnel axis angle in the axial plane (*p* = 0.008).

Several authors have recommended appropriate tunnel lengths. The ideal or minimal tunnel length remains unclear, but most surgeons anecdotally prefer to have 20 mm or more of graft to allow strong tendon healing to the bone within the tunnel. The lack of graft incorporation is a common cause of surgical failure [14]. Greis et al. reported that the length of a tendon placed within a bone tunnel influences tendon pullout strength, and advocated maximizing the tendon length inside the bone tunnels [22]. Previous cadaveric and clinical studies reported that the mean femoral tunnel length drilled through AM portals ranges from 15.7 − 34.2 mm [23, 24]. Moreover, extra length is required to flip and subsequently seat a suspensory fixation button device on the outside of the femoral cortex.

To attain adequate tunnel length with the AM portal technique and prevent posterior wall breakage, many researchers have investigated femoral tunnel orientation and intraosseous length changes with knee flexion angle. Basdekis et al. [20] and Bedi et al. [25] noted that increasing knee flexion increases the tunnel length and decreases the risk of posterior cortical breakage. Iyyampillai et al. suggested that femoral tunnel drilling with maximal knee hyperflexion in ACL reconstruction consistently produced tunnel lengths greater than 30 mm with no posterior wall fractures [26]. However, others have suggested that increased sagittal inclination or reduced guide wire axial angles have little effect on tunnel length [20, 25, 27]. Some have refuted the correlation of knee flexion angle with tunnel length because maximum flexion varies from one subject to another. In addition, tunnel lengths might be affected by patient height, weight, leg size, operative positioning, and larger lateral femoral condyle dimensions [14, 26].

However, previous studies have been based on fixed intra-articular tunnel orientation by drilling with a straight guide pin. We thought it was necessary to investigate tunnel configurations made using curved guides with a three-dimensional approach because it allows a certain degree of control over intra-articular tunnel orientation. Few previous studies have investigated the ideal tunnel axis angle for longer tunnel lengths [22, 28]. These studies were limited because they were experimental studies using a bone saw or cadaver, and the results are difficult to reproduce in a practical surgical situation. To our knowledge, no in vivo studies have been conducted with respect to the potential correlation between femoral tunnel length and guide pin entrance angle after anatomic ACLR with the TP technique.

Regression analyses confirmed our hypothesis that tunnel length is influenced by changes in the tunnel axis angle, and the AM and PL tunnels were significantly correlated with changes in the tunnel axis angle for each different plane.

For AM tunnels, multivariate regression showed that tunnel length had significant correlations with tunnel orientation in the sagittal and coronal planes (*p* = 0.022, *p* = 0.029, respectively) with high reliability (adjusted R^2^ = 0.801), while there was no significant correlation in the axial plane (*p* = 0.493). We interpreted this result as 1) longer tunnel lengths can be achieved when the sagittal tunnel angle is increased because outer orifice of the tunnel on the globular-shaped lateral condyle would be made further from the tunnel placement position, 2) the increased coronal tunnel angle provides tunnels with longer hypotenuses, and 3) there was no significant correlation in axial tunnel angle with tunnel length because the inner orifice of the AM tunnels is located in the posterior side of the anatomic ACL footprint, making it technically difficult to create a tunnel outlet oriented near the lateral epicondyle or the apex of lateral femoral condyle, due to abrupt tunnel bending angles. Contrary to the AM tunnels, for the PL tunnels, only the axial tunnel angle showed a significant correlation to tunnel length (*p* = 0.008, adjusted R^2^ = 0.700). We explained this result as 1) the PL tunnel can made easily in the outer orifice near the apex of the lateral femoral condyle, lateral epicondyle, and nearer to the lateral epicondyle, which would increase the axial tunnel angle and lengthen the tunnel, and 2) significant correlations with the tunnel axis angle in the sagittal and coronal planes were not identified due to restricted changes in the tunnel axis angle in the sagittal and coronal planes by its more intra-articular horizontal tunnel orientation even though the curved guide was used.

Recently, the CLANCY 42° curved guide (Clancy Anatomic Cruciate Guide System; Smith & Nephew, Andover, MA) has been utilized with the TP technique to access native femoral ACL insertion. This method achieves longer tunnel lengths with straight, rigid instrumentation that does not require hyperflexion, but is associated with the loss of visualization, fat pad ingress, poor arthroscopic inflow, inability to seat instrumentation, and the bending of rigid guide wires [14]. Andrew et al. noticed that commercially available flexible reamers and curved guides result in longer femoral interosseous tunnel lengths than those achieved with a straight guide pin [14, 29]. Furthermore, the rotational freedom of the intra-articular portion of a curved guide can assure a wide variability of tunnel angle orientation (Fig. 1) (video clip is available as a Additional file 1: Movie S1).


Additional file 1: Movie S1. Intentional rotary movement of intra-articular portion of a curved guide. The rotational freedom of the intra-articular portion of a curved guide can assure a wide variability of tunnel angle orientation. (MOV 101932 kb)


Our results provide a technical consideration for the acquisition of longer tunnel lengths, combined with the use of commercially available flexible reamer and curved guide systems. This study also indicates that three-dimensional tunnel orientations should be stressed for appropriate femoral tunnel length in anatomic ACL reconstruction.

This study has several limitations. At first, there may be a certain degree of variability in the AM and PL femoral tunnel center locations. However, every efforts were put to standardize the starting position of femoral tunnels according to anatomic landmarks of the lateral femur and the knee flexion angle, described in previous literature from our team [15, 24]. And the individual data in the present study has been controlled for patient factors and anatomic variables that may affect tunnel length, and this study was performed using 3D virtual models constructed by applying reverse engineering software with high reliability and accuracy. Several authors have stressed the limitations of two-dimensional radiographic assessment [30, 31]. Khalfayan et al. [32] included inadequate radiographic data as one of the exclusion criteria in their study. Sommer et al. [33] were concerned with tunnel invisibility on the tunnel view and inaccurate projection on the lateral view. Seconds, we are not able to be sure that all femoral tunnels in this study provide the best configuration of tunnels. However, our considerations at femoral tunneling should be common agreements to other surgeons for anatomic ACL reconstruction.

## Conclusion

Drilling at a higher angle in the coronal and sagittal planes in AM tunnels and at a higher angle in the axial plane in PL tunnels decreases the incidence of short femoral tunnels.

## Abbreviations

AAM: Accessory anteromedial
ACLR: Anterior cruciate ligament reconstruction
AM: Anteromedial
CT: Computed tomography
HTO: High tibial osteotomy
LPC offset distance: Lateral posterior condyle (LPC) AP size
M-L: From medial to lateral
PL: Posterolateral
TP: Transportal
